# The Intermolecular NOE Depends on Isotope Selection: Short Range vs Long Range Behavior

**DOI:** 10.1021/acs.jpclett.1c02253

**Published:** 2021-09-02

**Authors:** Philipp Honegger, Maria Enrica Di Pietro, Franca Castiglione, Chiara Vaccarini, Alea Quant, Othmar Steinhauser, Christian Schröder, Andrea Mele

**Affiliations:** † Department of Systems Biology, Harvard Medical School, 200 Longwood Avenue, Boston, Massachusetts 02115, United States; ‡ Department of Computational Biological Chemistry, University of Vienna, Währinger Straße 17, 1090 Vienna, Austria; § Department of Chemistry, Materials and Chemical Engineering “G. Natta”, Politecnico di Milano, Piazza L. da Vinci 32, 20133 Milano, Italy; ∥ CNR-SCITEC Istituto di Scienze e Tecnologie Chimiche, Via A. Corti 12, 20133 Milano, Italy

## Abstract

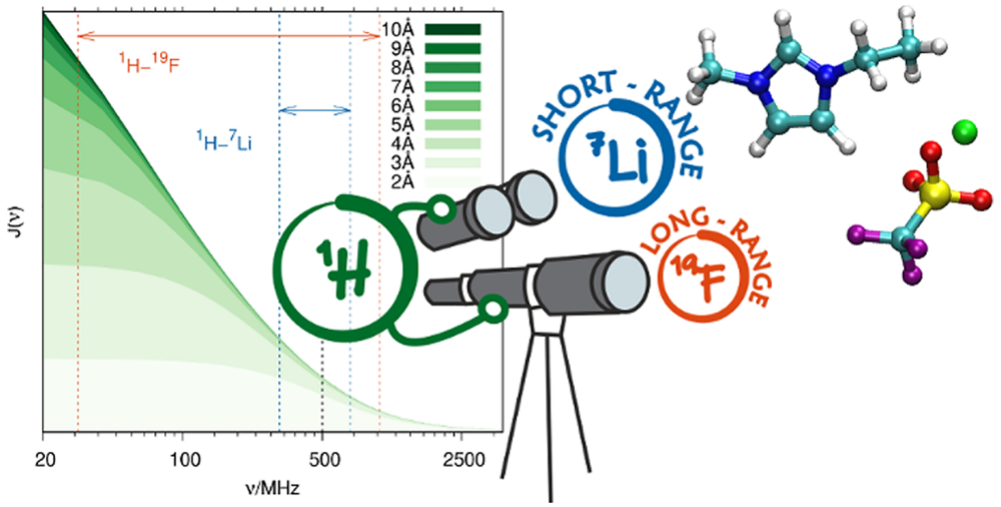

The nuclear Overhauser effect (NOE) is a powerful tool in molecular structure elucidation, combining the subtle chemical shift of NMR and three-dimensional information independent of chemical connectivity. Its usage for intermolecular studies, however, is fundamentally limited by an unspecific long-ranged interaction behavior. This joint experimental and computational work shows that proper selection of interacting isotopes can overcome these limitations: Isotopes with strongly differing gyromagnetic ratios give rise to short-ranged intermolecular NOEs. In this light, existing NOE experiments need to be re-evaluated and future ones can be designed accordingly. Thus, a new chapter on intermolecular structure elucidation is opened.

Ionic liquids (ILs) continuously attract interest in their applications and the still open issues on their fundamental knowledge.^1^ One of their most fascinating aspects is the so-called ”nanostructural organization” of polar and apolar domains,^2,3^ whose formation, nevertheless, does not lead to phase inhomogeneities or phase separation. Such a paradigmatic feature points out that for ILs and more generally for ”soft matter”, the nanoscopic size of the intermolecular structures cannot be observed optically. Instead, they need to be probed by electromagnetic radiation providing indirect information on molecular structure and processes. Since the interpretation of such spectral features is often nontrivial and may lead to contradicting viewpoints, it can be augmented by molecular dynamics (MD) simulations which serve as a mathematical microscope into the atomistic world.^4−6^

The tremendous synergy of spectroscopic methods and MD simulations is the charming toolkit for understanding interactions in liquid media leading to a particular structure and corresponding physicochemical properties. In 1995, the pioneering NMR paper by Osteryoung^7^ first showed the potential of the nuclear Overhauser effect (NOE) as a detection tool of intermolecular contacts in liquids that opens the route to NOE-based investigations on the local structure of ILs.^8^ The unique role of NOE in the large repertoire of NMR techniques is related to the fact that NOE depends on spatial dipolar interactions of nuclei rather than chemical connectivity via chemical bonds. Consequently, NOE is a powerful tool to characterize the structure, interaction, and dynamics in liquids.^9,10^ On the level of molecular processes, the temporal evolution (randomization rate) of the NOE is described by the time correlation function (TCF)
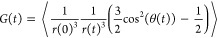
1with *r⃗*(*t*) as the vector connecting the two interacting nuclei *I* and *S* at time *t*, and θ(*t*) is the angle swept by this vector during timespan *t*. In the frequency domain, this corresponds to the spectral density function (SDF)
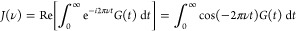
2with Re extracting the real part of the Fourier transformation. Cross-relaxation rates are then calculated using Larmor frequencies ν_*I*_ = γ_*I*_ ν and ν_*S*_ = γ_*S*_ ν:
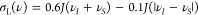
3The intramolecular NOE has become a standard technique in molecular structure elucidation. The distance of the interacting nuclei *r⃗* is constant except for molecular vibrations and segmental motion and depends only on molecular rotation. This yields a strict 1/*r*^6^ distance dependence that ensures that a meaningful NOE can only occur up to 4–5 Å.^11^ This well-known distance dependence mediated by dipole-coupling can be understood from eq 1. The situation changes for intermolecular NOEs, where the interacting spins are not located on the same molecule:^12^

- A reference spin interacts with *many* surrounding spins. Instead of one internuclear distance, there is a distribution of distances, known as the radial distribution function (RDF). We are primarily interested in the contact shell surrounding a reference molecule. The molecules beyond form the bulk (Figure S1). The number of partner spins increases by an order of *r*^2^ with increasing distance.
- The greater the distance between two interacting spins, the more time the spin-joining vector needs to randomize its length and orientation. The randomization time also increases by order of *r*^2^ (Figure S2).
- Summing up over all spherical distance shells *r* adds an order of *r*.

Thus, in the extreme case, using model theory, Halle predicted a long-ranged intermolecular NOE decaying by an order of 1/*r*.^12^ MD simulations expanding on those models showed a somewhat more beneficial but still long-ranged behavior between 1/*r* and 1/*r*^3^.^13^ Structural short-ranged information is present in intermolecular NOEs too but buried by unspecific magnetization transfers between the reference molecule and a multitude of distant bulk molecules.^14^

The lower the frequency of the spectral density function *J*(ν), the more long-ranged the NOE becomes. As pointed out by Weingärtner and co-workers in their seminal paper, the interaction range depends on the spectrometer frequency.^15^

The approach proposed by Castiglione et al. in ref (16) and treated in this work seeks to select general cases in which the short-distance based interpretation on NOE still holds but, at the same time, does not contradict the general theory of intermolecular cross-relaxation. The cross-relaxation rate σ_L_ is a linear combination of the high-frequency part *J*(ν_*I*_ + ν_*S*_) and the low-frequency part *J*(|ν_*I*_ – ν_*S*_|) of the SDF. Indeed, the long-ranged contributions of *J*(ν →0) can be made negligible by selecting isotopes maximizing the frequency difference |ν_*I*_ – ν_*S*_| = |(γ_*I*_ – γ_*S*_)·ν|.

In this instance, we propose that the heteronuclear NOE of ^1^H (γ_H_ = 42.577 MHz T^–1^) and ^7^Li (γ_Li_ = 16.546 MHz T^–1^) is of a shorter range than the one between ^1^H and ^19^F (γ_F_ = 40.078 MHz T^–1^). This theory was tested by O’Dell and co-workers using a combination of MD simulations and quantitative HOESY analysis.^17,18^ The elegant (yet not straightforward) fit of suitably normalized HOESY build-up curves with a modified expression including both the longitudinal relaxation times and diffusion coefficients allows for the precise calculation of absolute intermolecular cross-relaxation rates and their comparative use between different ionic liquids, concentrations, or temperatures.^19,20^

The following calculations are based on a 500 MHz ^1^H NMR spectrometer (cf. eq 3):

- In the homonuclear extreme case, *J*(2ν) and *J*(0) contribute, e.g., 2ν = 1000 MHz for ^1^H. The high-frequency part is short-ranged but the low-frequency part is long-ranged, as can be seen in the top panel of Figure 1, rendering the ^1^H–^1^H NOEs long-ranged.
- The same is true for ^1^H–^19^F NOEs. The gyromagnetic ratios are similar (ν_*I*_ + ν_*S*_ = 970 MHz and |ν_*I*_ – ν_*S*_| = 30 MHz) resulting in similar problems as the homonuclear case.
- For ^1^H–^7^Li NMR, the gyromagnetic ratios differ significantly; hence both terms at ν_*I*_ + ν_*S*_ = 694 MHz and |ν_*I*_ – ν_*S*_| = 306 MHz draw from a similar part of the SDF and avoid the low-frequency part, prospecting a more beneficial short-ranged behavior.

The less similar the gyromagnetic ratios, the higher is the low-frequency |ν_*I*_ – ν_*S*_|, avoiding the long-ranged low-frequency SDF limit *J*(ν → 0).

**Figure 1:**
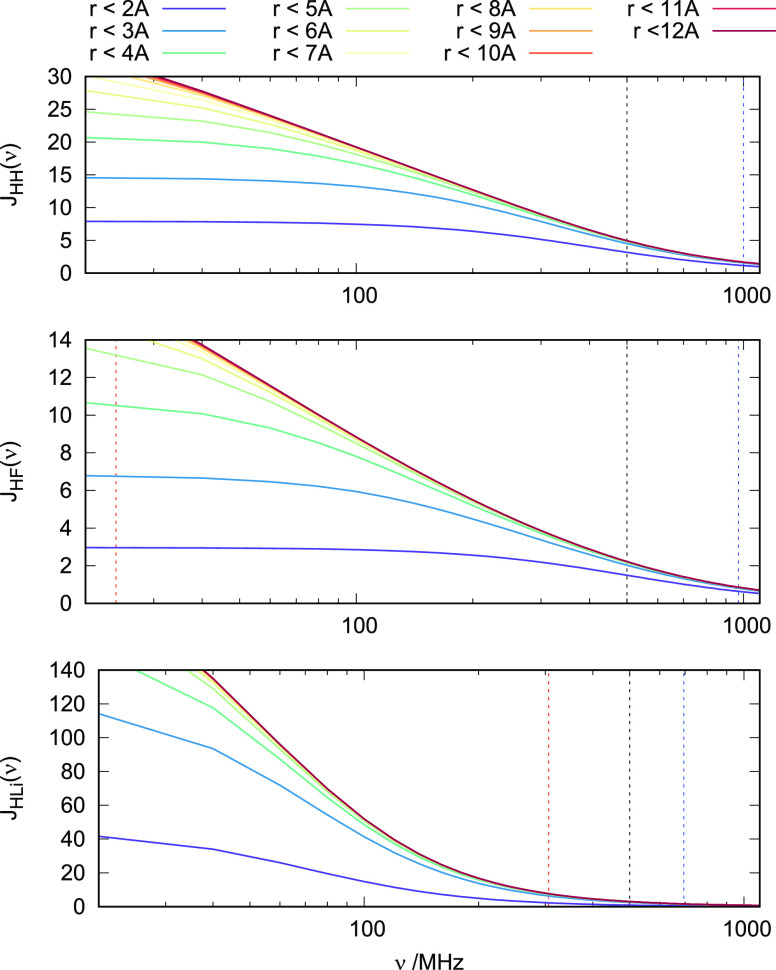
SDF *J*(ν) of the H8–H8 (top), H8–F (middle), and H8–Li (bottom) spin pairs, resolved into cumulative contributions. The lower the frequency, the more long-ranged the *J*(ν) becomes. Explicitly marked frequencies are the following: spectrometer (black), high-frequency contribution *J*(ν_*I*_ + ν_*S*_) (blue), low-frequency contribution *J*(ν_*I*_ – ν_*S*_) (red, beyond range for ^1^H–^1^H)

In this proof-of-concept study, we perform semiquantitative experimental NOE measurements of the ionic liquid/salt solution 0.9 1-ethyl-3-methylimidazolium [C_2_MIm]·0.1 Li·1.0 triflate [OTf] (Scheme 1). The simple fit of the HOESY build-up curves with the fundamental expression derived by Solomon equations^11,21^ gives relative cross-relaxation rates, with no need for elaborate normalization or fitting procedures. Still, we demonstrate that these relative values do reflect the heteronuclear proximity when interpreted bearing in mind the isotope dependency of short- and long-range contributions. This joint theoretical and experimental validation represents then a first step for some ”nuts and bolts” guidelines for the interpretation of intemolecular NOEs for non-NMR-specialists; thus it is of broad interest for the wider chemist community.

**Scheme 1:**
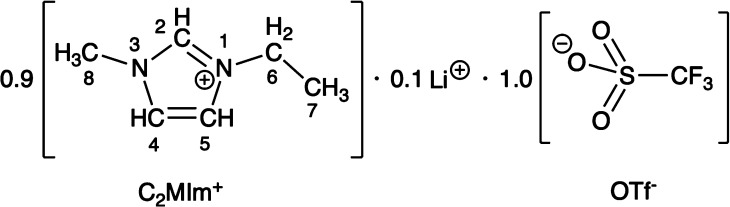
Chemical Structure of the IL/Salt Mixture

Our interpretations of the experimental NOEs are supported by an MD simulation. In contrast to preceding works, we modeled the molecules as both fully atomistic and polarizable. Nonpolarizable ILs exhibit exaggerated directed electrostatic interactions. In actuality, electronic charge distributions are flexible upon molecular contact, and thus ILs are less viscous,^22^ necessitating polarizable force fields to allow for realistic pair diffusion dynamics important to the NOE. Furthermore, we calculate the NOE directly from the trajectory instead of using models such as hard-sphere free diffusion,^23^ spin eccentricity,^24^ or the monoexponential Bloembergen–Purcell–Pound equation,^25^ thus avoiding their intrinsic model assumptions. The TCF *G*(*t*) is obtained from nuclear positions in the trajectory and transformed into the SDF *J*(ν), necessitating a trajectory length of at least (100 MHz)^−1^ = 10 ns (see Supporting Information for details).

We found cross-peaks of all C_2_MIm^+^ protons interacting with the CF_3_ group of OTf^–^ (Figure S3a) and Li^+^ (Figure S3b). The individual signal intensities are proportional to the magnitude of the given cross-relaxation and hint about the intensity of the mutual interaction. Figure 2 (top row) presents the NOEs of the neat IL [C_2_MIm][OTf] and solution 0.9[C_2_MIm]·0.1Li·1.0[OTf]. Some considerations can be drawn about the trends in experimental NOE values:

- The short-ranged ^1^H–^7^Li HOESY reflects specific Li-cation interactions. Li preferentially interacts with N–CH_3_ (H8) and roughly equivalently with all other proton sites. The alkyl chain of imidazolium-based ILs is known to form hydrophobic domains, excluding charged functional groups.^13,26^ This can be seen in the ^1^H–^7^Li RDFs of the MD simulation as well (Figure S1): The H8–Li atom pair forms a higher peak at a short distance (≈3–4 Å) than either H6–Li and H7–Li.
- ^1^H–^19^F HOESY contains considerable contributions from the bulk. The most intense correlations of the anion are with the alkyl protons. Yet this behavior is neither chemically intuitive nor justified by the RDFs (Figure S1). The H–F NOEs are contaminated with unspecific long-ranged contributions and thus arbitrarily unreliable. In this respect, we observe that there is no considerable variation between the neat and Li-loaded samples.

**Figure 2:**
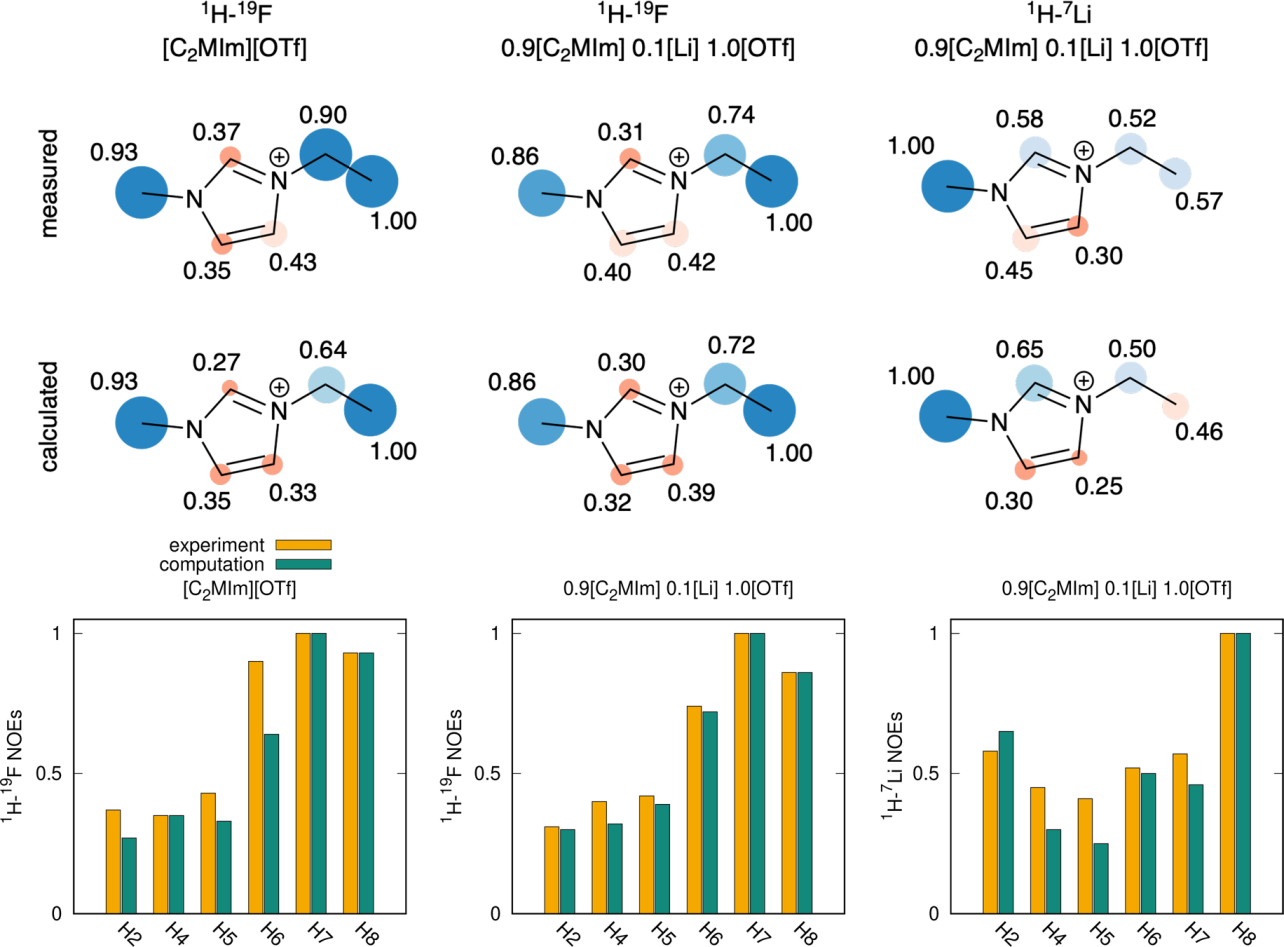
Top: Experimental (upper row) and computational (bottom row) C_2_MIm^+^-F and C_2_MIm^+^-Li NOEs for the neat IL [C_2_MIm][OTf] and its mixture with [Li][OTf], normalized to the most intense NOE signal per molecule. Experimental parameters: *T* = 298 K, mixing times were 900 ms (C_2_MIm^+^-F) and 1200 ms (C_2_MIm^+^-Li). Bottom: Comparison of experimental and simulated NOEs.

For a semiquantitative analysis of HOESY cross-peaks, integrated volumes were corrected by a factor *N*_*I*_*N*_*S*_/(*N*_*I*_ + *N*_*S*_), with *N*_*I*_ the number of ^1^H and *N*_*S*_ the number of ^19^F or ^7^Li nuclei contributing to the observed NOE signal.^16,27−32^ The corresponding corrected and normalized NOE build-up curves obtained from 29 ^1^H–^19^F and 23 ^1^H–^7^Li spectra, at increasing mixing time, are displayed in Figures 3a and S4. As expected, from 20 ms to 700–900 ms, a linear increase is observed in ^1^H–^19^F build-up curves, then a maximum is reached, and an exponential decay is observed afterward. Similar behavior is seen in ^1^H–^7^Li HOESY, with the maximum shifted to 1.2 s.

**Figure 3:**
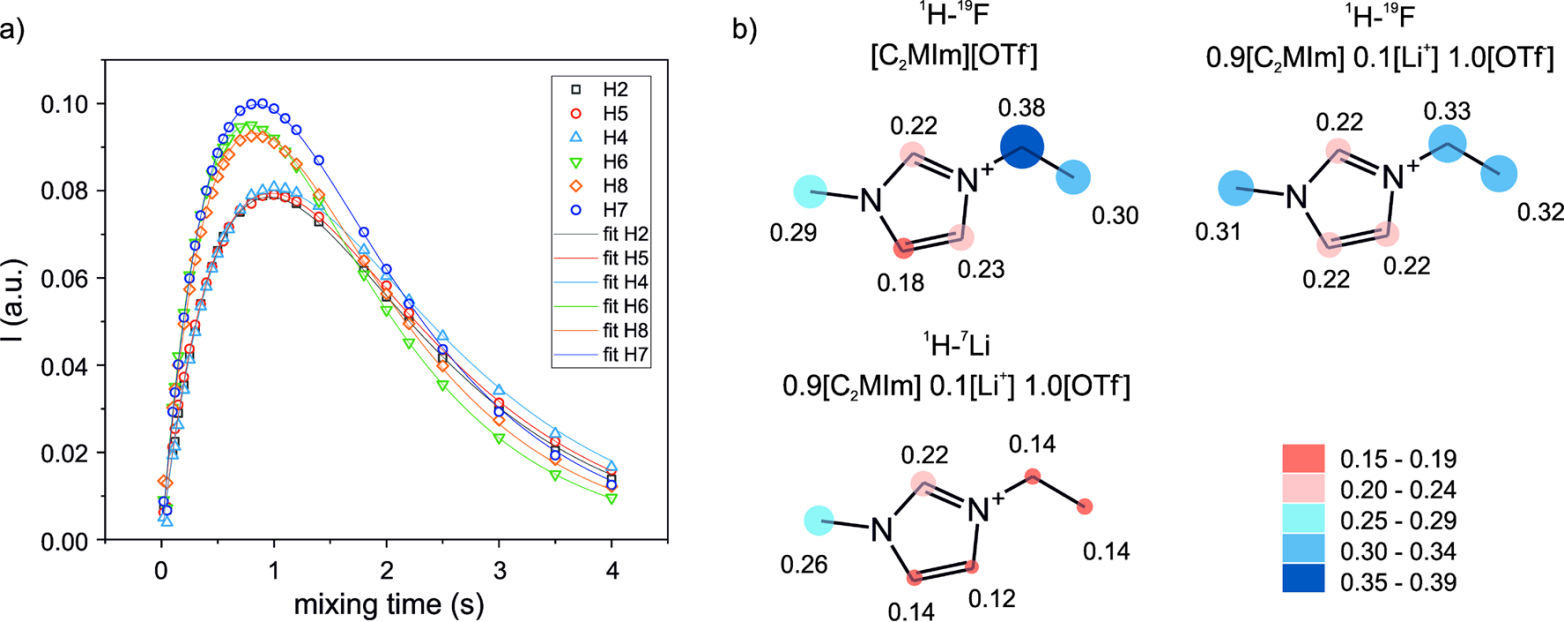
(a) ^1^H–^19^F HOESY build-up curves for the mixture 0.9[C_2_MIm]·0.1 Li·1.0[OTf] and corresponding fit using eq 4. (b) Cross relaxations obtained from the fit with eq 4 of the corrected and normalized ^1^H–^19^F and ^1^H–^7^Li NOEs observed for the neat IL and its mixture with [Li][OTf].

All curves were fitted using an exponential function derived from the fundamental Solomon equations,^11,21^ using *R* (total longitudinal relaxation rate constant) and σ_*IS*_ as fit-able parameters:
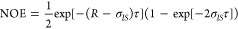
4Figure 3b displays the cross relaxations σ_*IS*_ obtained by fitting. Findings are in agreement with NOEs for both ^1^H–^19^F and ^1^H–^7^Li interactions. As a result of the correction, the difference in intensity between the interactions at the different sites is reduced. For instance, looking at the ^1^H–^19^F cross relaxations, those with the alkyl protons are still dominating, but the differences with the imidazolium protons are less significant. Similarly, the ^1^H–^7^Li cross-relaxation at N–CH_3_ site has the highest value but less marked difference to the other protons.

The computational ^1^H–^19^F and ^1^H–^7^Li NOEs are shown Figure 2 (second row). We find a reasonably good match between experimental and simulated NOEs (Figure 2, bottom). The most significant observed difference is in the ^1^H–^7^Li NOE of the imidazolium H4 and H5 protons in the mixture and the ^1^H–^19^F NOE of the CH_2_ protons in the neat IL. Since the overall trend of NOEs is faithfully reproduced, these four diverging values out of 33 spin pairs are not systematic and are likely local artifacts. The polarizabilities used in this work are more or less a function of the hybridization and the number of attached protons but take less into account the immediate chemical environment. Nevertheless, the emerging induced dipoles of these carbons based on these polarizabilities react individually to their local environment. Of course, an exact quantum-mechanical determination of the polarizabilities is also possible^33,34^ and leads to slight variations in the respective carbon polarizabilities and hence slightly different induced dipoles, but using new polarizabilities would require a complete reparametrization of the polarizable force field. The one applied in this work, however, has already proven to reproduce experimental NMR results.^35^

The reasonable agreement with the experimental values validates the accuracy of the MD simulation; thus the computational NOE calculations can be used to decompose observable sum spectra into different components. We dissect σ_L_(ν) into contributions from spin pairs at different distances,
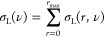
5Figures 4 (top) and S5 display the convergence to the experimentally observable σ_L_(ν).

**Figure 4:**
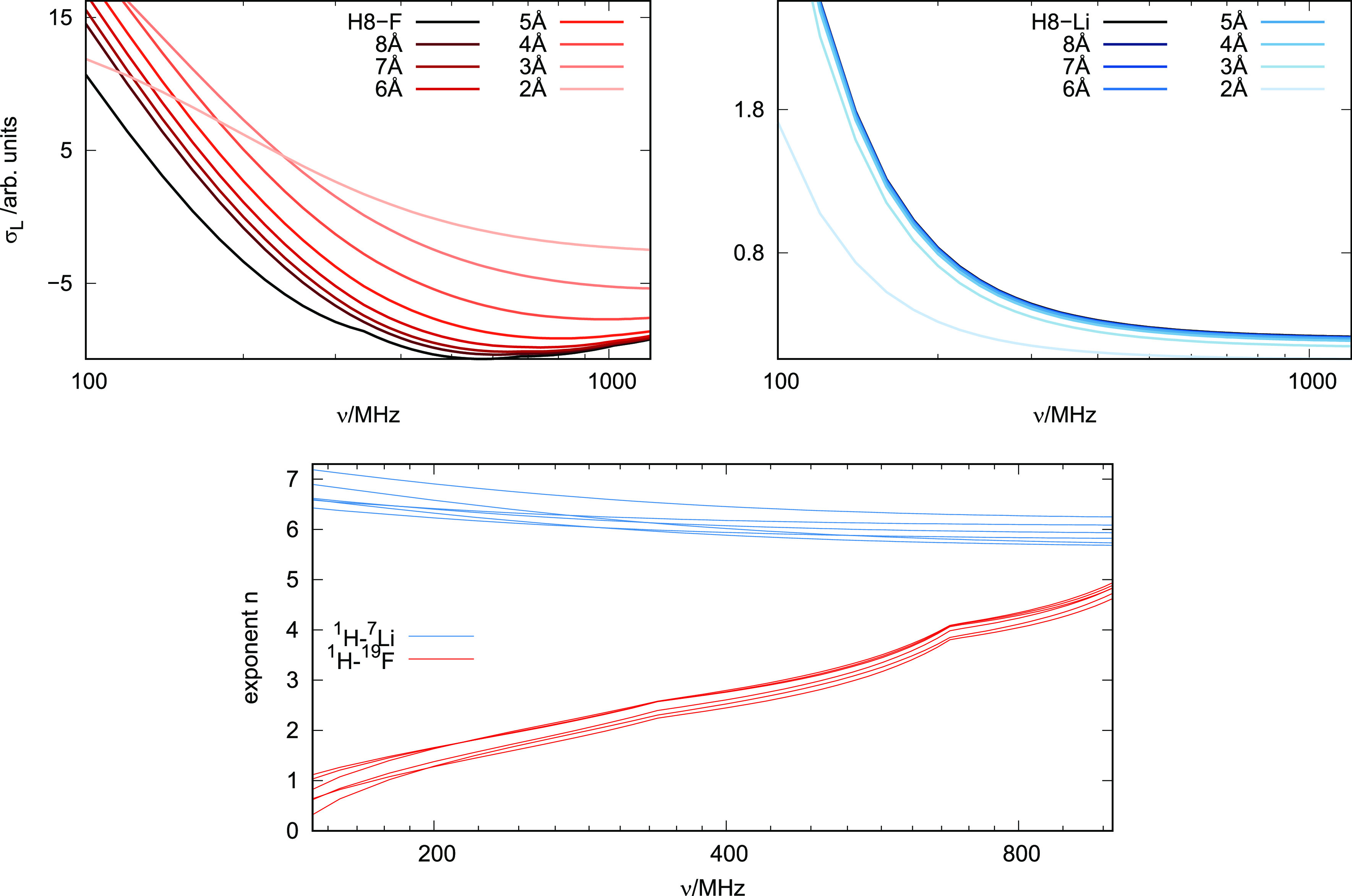
Cumulative contributions of shell-resolved cross-relaxation rates σ_L_(*r*,ν) of the H8–F spin pairs (left) and the H8–Li spin pairs (right). σ_L_ converges faster for ^1^H–^7^Li spin pairs than ^1^H–^19^F spin pairs. Bottom: Interaction range of the H–F spin pairs (red) and the H–Li spin pairs (blue), represented by the exponent of the decay law 1/*r*^*n*^.

The ^1^H–^19^F contributions converge at larger distances than ^1^H–^7^Li contributions, meaning the latter are barely affected by spin interactions with the bulk. In addition, the frequency dependence of the ^1^H–^19^F spin pairs is more pronounced: The spacing between the curve bundles varies from wide (low ν, long-ranged) to small (high ν, short-ranged). In order to quantify the changed range dependence, we fitted the spatially resolved σ_L_(*r*,ν) to a 1/*r*^*n*^ law, shown in Figure 4(bottom). The H–Li spin pairs show a relatively consistent short-ranged 1/*r*^6^ distance dependence. The range of H–F spin-pairs is long and additionally depends on the spectrometer frequency.

In summary, this contribution follows up the 2013 milestone paper by Gabl, Steinhauser, and Weingärtner, who introduced the fundamental concept that the structural information from intermolecular NOE is severely affected by the Larmor frequency of the interacting nuclei: “frequency does matter”,^15^ strongly discouraging the usage of intermolecular NOEs for structure determination in the chemist’s community. Their work studied a ^1^H–^19^F NOE.

Here we demonstrate how gyromagnetic ratios of interacting nuclei determine the intermolecular NOE range. The larger their difference, the larger the Larmor frequency difference |ν_*I*_ – ν_*S*_| becomes, avoiding the long-ranged low-frequency limit. We studied the IL electrolyte 0.9[C_2_MIm]·0.1 Li·1.0[OTf] as a prototypical example with at least two remarkable outcomes:

- The good agreement between HOESY measurements and calculations validates the correctness of the computational results.
- Computational signal decomposition confirms that the ^1^H–^19^F signal contains significant interactions with the bulk. In contrast, the ^1^H–^7^Li signal converges at small distances and is thus specific.

Our work provides experimentalists with a clear-cut interpretation tool for the structural use of intermolecular NOE: Proper selection of isotopes with differing gyromagnetic ratios overcomes the fundamental long-ranged limitation of intermolecular NOEs and provides intermolecular structural information.

Finally, the long-range interpretation of intermolecular NOEs should not prevent chemists from measuring them, as their interpretation adds value to other structural methods for assessing mesoscopic order, such as WAXS, SAXS, or SANS. As previously shown by some of us, the joint use of NOEs, scattering techniques, and MD simulations is a powerful investigating tool for nanostructured liquids.^36^

## Supporting Information Available

Theory and experimental and computational methods in detail (PDF)
