# Supplementary material for: The Intermolecular NOE Depends on Isotope Selection: Short Range vs Long Range Behavior
Source: J Phys Chem Lett. 2021 Sep 2;12(35):8658–63. doi: 10.1021/acs.jpclett.1c02253 (PMC8436203; doi:10.1021/acs.jpclett.1c02253)
Supplement: Supplementary file 1 — jz1c02253_si_001.pdf [file jz1c02253_si_001.pdf]

Supporting Information for: The Intermolecular NOE Depends on Isotope Selection: Short Range vs. Long Range Behavior

Philipp Honegger,[†,‡,∥] Maria Enrica Di Pietro,[¶,∥] Franca Castiglione,[*,¶] Chiara Vaccarini,[¶] Alea Quant,[‡] Othmar Steinhauser,[‡] Christian Schröder,[*,‡] and Andrea Mele[¶,§]

†*Department of Systems Biology, Harvard Medical School, 200 Longwood Avenue, Boston MA 02115, United States*

‡*Department of Computational Biological Chemistry, University of Vienna, Währinger Straße 17, 1090 Vienna, Austria*

¶*Department of Chemistry, Materials and Chemical Engineering "G. Natta", Politecnico di Milano, Piazza L. da Vinci 32, 20133 Milano, Italy*

§*CNR-SCITEC Istituto di Scienze e Tecnologie Chimiche, Via A. Corti 12, 20133 Milano, Italy*

∥*These authors contributed equally*

E-mail: franca.castiglione@polimi.it; christian.schroeder@univie.ac.at

1 Theory

The nuclear Overhauser effect describes a magnetization transfer between two different nuclei with spins I and S via a dipole-coupled mechanism. Its dynamics can be described by the first-order differential equations,[1]

$$\frac{d\langle I_z\rangle}{dt} = \rho_{II}\langle I_z\rangle + \sigma_{IS}\langle S_z\rangle \quad (1)$$
$$\frac{d\langle S_z\rangle}{dt} = \sigma_{SI}\langle I_z\rangle + \rho_{SS}\langle S_z\rangle \quad (2)$$

a special case of Redfields relaxation theory[2] where I_z and S_z represent the longitudinal magnetization of spins I and S. The structural information of the system studied is contained in the self- and cross-relaxation rates ρ and σ modulating the transfer rate, sometimes referred to as the 'lattice' or, more descriptively, 'chemical environment'. These relaxation rates can be measured experimentally. They depend on the spectrometer frequency ν and the isotope-specific Larmor frequencies $\nu_I = \nu \cdot \gamma_I$ and $\nu_S = \nu \cdot \gamma_S$:

$$\sigma_L^{\text{NOESY}} = 0.6 J_{IS}(\nu_I + \nu_S) - 0.1 J_{IS}\big(\mid \nu_I - \nu_S \mid\big) \quad (3)$$
$$\sigma_R^{\text{ROESY}} = 0.3 J_{IS}(\nu_I) + 0.2 J_{IS}\big(\mid \nu_I - \nu_S \mid\big) \quad (4)$$

where σ_L^{NOESY} and σ_R^{ROESY} refer to the laboratory- and the rotation frame cross relaxation rate. $J(\nu)$ is the spectral density function (SDF) which contains the information of the dipole-coupled system. It can be obtained via real-part Fourier transform as

$$J_{IS}(\nu) = \text{Re}\Big[\int_0^\infty e^{-i2\pi\nu t} G_{IS}(t)\, dt\Big] = \int_0^\infty \cos(-2\pi\nu t) G_{IS}(t)\, dt \quad (5)$$

with $G_{IS}(t)$ as the time correlation function (TCF), $G_{IS}(t) = \mathrm{Tr}\left\langle \overleftrightarrow{T}_{IS}(0)\cdot \overleftrightarrow{T}_{IS}(t)\right\rangle$, of the dipole-dipole tensor trace involving the two interacting spins:

$$\overleftrightarrow{T}_{IS}(t) = \frac{1}{r_{IS}(t)^3}\left(\frac{3\vec{r}_{IS}(t) : \vec{r}_{IS}(t)}{r_{IS}(t)^2} - \overleftrightarrow{E}\right) \tag{6}$$

where $\vec{r}_{IS}(t) = \vec{r}_I(t) - \vec{r}_S(t)$ is the vector joining the interacting nuclei I and S at time t, $\overleftrightarrow{E}$ is the unity matrix and the colon marks the dyadic product of two vectors.

The TCF of two spins I and S can be rewritten as

$$G_{IS}(t) = \left\langle \frac{1}{r_{IS}(0)^3}\frac{1}{r_{IS}(t)^3}\left(\frac{3}{2}\cos^2(\theta_{IS}(t)) - \frac{1}{2}\right)\right\rangle \tag{7}$$

where $\theta_{IS}(t)$ is the angle swept by the spin-joining vector $\vec{r}_{IS}$ during timespan t. In this form, the temporal evolution of the NOE becomes clear: The dynamics of the TCF depend on the randomization rate of the spin-joining vector $\vec{r}_{IS}$. This vector can be either randomized by changing the internuclear distance (mutual diffusion towards or away from each other) or altering the internuclear orientation. Of these two, translational mutual diffusion is the prominent dynamics in the intermolecular case.[3]

In principle, structural information is present in the amplitude $G_{IS}(t = 0)$ of the TCF:[4]

$$G_{IS}(0) = 4\pi\rho_{IS}\int_0^\infty r^2\, g_{IS}(r_{IS})\frac{1}{r^6}\, dr \tag{8}$$

with ρ_{IS} as the number density of particles in a volume and $g_{IS}(r_{IS})$ as the radial pair distribution function (RDF) of spins I and S. In essence, this is analogous to the calculation of a coordination number, but weighed by a decay term $1/r^6$. From this, we can infer that the SDF at $\nu = 0$ is the product of the amplitude $G_{IS}(0)$ (structural information) and the relaxation time τ_{IS} (dynamics information):

$$J_{IS}(0) = \int_0^\infty G_{IS}(t)\, dt = G_{IS}(0)\int_0^\infty \frac{G_{IS}(t)}{G_{IS}(0)}\, dt = G_{IS}(0)\cdot\tau_{IS} \tag{9}$$

Experimentally, we have neither access to the TCF amplitude $G(0)$ nor the zero-frequency SDF $J(0)$. Instead, spectrometer frequency ν (*e.g.* 500 MHz for ^{1}H) determines the part of the SDF $J(\nu)$ and of the correlation function $G(t)$ obtainable by experiment, inherently influenced by the dynamics of the sample.

2 Methods

2.1 Experimental

The ionic liquid 1-ethyl-3-methylimidazolium triflate (99%) and the salt lithium trifluoromethanesulfonate (96%) were purchased from Iolitec and Sigma Aldrich, respectively. A mixture was prepared by combining IL and salt in a 0.9:0.1 molar ratio. After proper stirring, both the neat IL [C_2MIm][OTf] and the mixture 0.9 [C_2MIm] · 0.1 [Li^+] · 1.0 [OTf^-] were dried under *vacuum* and transferred in 5 mm NMR tubes. The tubes were equipped with a capillary containing DMSO-d_6 and immediately flame-sealed after transferring the samples. The absence of a detectable water signal in the NMR spectra indicated that the water content is negligible.[5] NMR experiments were carried out at 298 K without sample spinning with a Bruker NEO 500 console (11.74 T) equipped with a direct observe BBFO (broadband including fluorine) iProbe and a variable-temperature unit. The instrument was carefully tuned, shimmed, and the 90° pulses calibrated. ^{1}H-^{19}F and ^{1}H-^{7}Li HOESY experiments were acquired using the phase sensitive echo-antiecho pulse sequence (hoesyetgp in the Bruker library) with observation on ^{1}H and ^{7}Li channel, respectively. 29 spectra were recorded using 4 transients over 2048 (t_2) × 128 (t_1) complex data points for ^{1}H-^{19}F HOESY and 23 spectra using 4 transients over 2048 (t_2) × 256 (t_1) complex data points for ^{1}H-^{7}Li HOESY. In all cases, 32 dummy scans and a 9s-long relaxation delay were used. The range for the mixing time was 20 ms - 4 s for ^{1}H–^{19}F experiments and 50 ms - 6 s for the ^{1}H-^{7}Li experiments. All HOESY data sets were processed by applying a sine squared window function in both dimensions (SSB = 2) and zero-filling to 2048 (t_2) × 512 (t_1) prior to the Fourier transform. The cross-peak integrals were measured using the Bruker software. For a semi-quantitative interpretation of the data, the peak volume of each cross-peak has been corrected by dividing the original integral by a correction factor taking into account the contribution of the number of spins, $N_I \, N_S/(N_I + N_S)$, with N_I the equivalent spins I (^{1}H) and N_S the equivalent spin S (^{19}F or ^{7}Li).[6–12]

2.2 Computational

2.2.1 Molecules and force field

In contrast to our preceding IL-NOE study,[4] the molecules (C_2MIm^+, Li^+ and OTf^-) are modeled as fully atomistic to accurately represent the internuclear distances of spin-bearing ^1H, ^7Li and ^{19}F nuclei. In addition, the simulations are fully polarizable modelled by Drude oscillators. Non-polarizable simulations of ILs exhibit too directed and inflexible charges and hence too slow dynamics.[13] They do no capture the natural molecular dynamics caused by flexible charge distributions of net charged molecules.[14,15]

The trajectory of [C_2MIm][OTf] was reused from ref. 16. The trajectory of 0.9[C_2MIm^+]·0.1[Li^+]·1.0 [OTf^-] was produced analogously: The molecules were parametrized using the force field creation tool GAAMP,[17] CGenFF output[18,19] was refined with quantum-chemical data. The molecules were rendered polarizable by attaching Drude particles to all non-hydrogen atoms (mass = 0.2 amu, harmonic bond constant = 500 kcal/mol Å^2).

The non-hydrogen atoms and their attached Drude particle form an atomic dipole and account for induced dipole-induced dipole attraction. Hence the Lennard-Jones attraction of the polarizable atoms had to be reduced. We used a λ-scaling correction introduced by Vlugt *et al.*[20] and chose a scaling factor of $\lambda = 0.4$ in accordance to previous C_2MIm^+-based IL MD studies which accurately reproduced experimental dielectric spectra[16] and NMR field cycling dispersion curves.[21] Similar scaling approaches are reported by Pádua and co-workers.[22]

2.2.2 Equilibration and simulation

All subsequent fully atomistic, polarizable MD simulations were carried out with CHARMM.[23] The system comprises 900 molecules of C_2MIm^+, 100 ions of Li^+ and 1000 molecules of OTf^-.

The initial intermolecular geometry of the simulation box was generated using PACKMOL[24] followed by energy minimization via 1000 steps of a steepest-descent algorithm. The system was then equilibrated as an isothermal-isobaric NpT ensemble (T = 300K, p = 1

atm) with periodic boundary conditions until the simulation box length converged to its equilibrium extent.

The trajectory was produced as an isothermal-isochoric NVT ensemble with periodic boundary conditions and a dual Nose-Hoover thermostat[25,26] to keep the temperature of the atoms at 300 K and the temperature of the Drude particles at 1 K. The SHAKE algorithm was applied to covalent bonds involving hydrogen atoms to constrain high-frequency vibrations. This allowed for a time step of 0.5 fs, a write frequency of 50 fs, and a total trajectory length of 20 ns. Electrostatic interactions were calculated using the Particle-Mesh Ewald method[27,28] with conducting boundary conditions ($\kappa = 0.41$ Å^{-1}).

2.2.3 Post-production analysis

The spectral features of the simulation were calculated using a Python3 program introduced in ref. 21. The CHARMM trajectory was read into the program with the MDAnalysis module.[29,30] The first 5000 ps of the trajectory were discarded to exclude possible slow equilibration artifacts.

The trajectory was unfolded to remove toroidal coordinate jumps and allow for the natural diffusive motion of the molecules crucial to the dipole-coupled TCF. The time series of all spin-pairs was recorded ($1000 \times 11 + 900 \times 3 + 100 \times 1$ spins, totalling 95,226,900 pairs; 120 unique pairs by atomic identity; 36 unique pairs by chemical shift; further divided into 120 distance bins each) and correlated. Since the computational effort is considerable, the runtime performance was enhanced using Cython classes and functions and further accelerated by C++ implementations where possible. The SDFs were calculated from the TCFs with the numpy.fft library.

The RDFs $g(r)$ were obtained by MDAnalysis based python scripts. Their respective decomposition into first, second and bulk solvation shell (see Fig. 1) was done by Voronoi tesselation which was performed using the Voro++ library.[31]

3 Supplementary figures

3.1 Structure

The RDF between two particle sets is defined as:

$$g(r_{ij}) = \frac{1}{4\pi N_i \rho_j} \sum_{i=1}^{N_i} \sum_{j=1}^{N_j} \frac{\delta(\mid \vec{r_i} - \vec{r_j} \mid)}{\mid \vec{r_i} - \vec{r_j} \mid^2} \tag{10}$$

with N_i and N_j as the particle counts, $\rho_j = N_j/V$ as the number density and δ as the Dirac delta function. Fig. 1 presents the RDF $g(r_{ij})$ of all C_2MIm^+-Li and C_2MIm^+-F pairs, further divided into shells via Voronoi tesselation,[32] a purely geometrical, parameter-free procedure.[33–35] The first shell, often referred to as the contact shell, is of particular interest to the structure. The second shell is characterized by the transition to the bulk formed by the third and subsequent shells. Voronoi tesselation is a more reliable descriptor of particle neighborhood for anisotropic molecules than radial shells.[36]

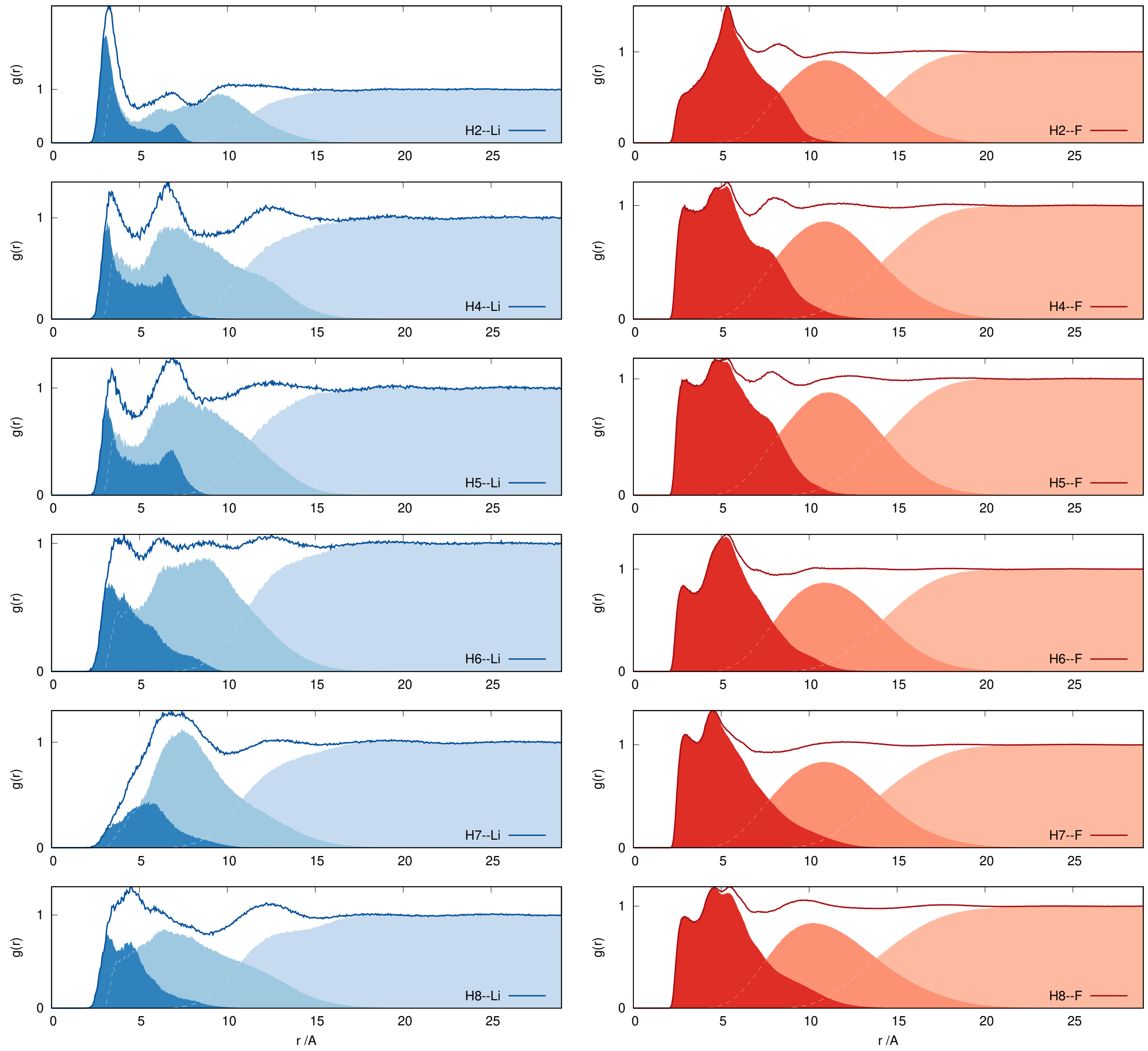

Figure 1: Pair distribution functions $g(r)$ of C_2MIm^+ protons and Li^+ (left-hand side) or F in OTf^- (right-hand side). The solid line represents the total RDFs, the filled curves indicate the contributions of the first, second and third solvation shell (left to right).

3.2 Relaxation times and amplitudes

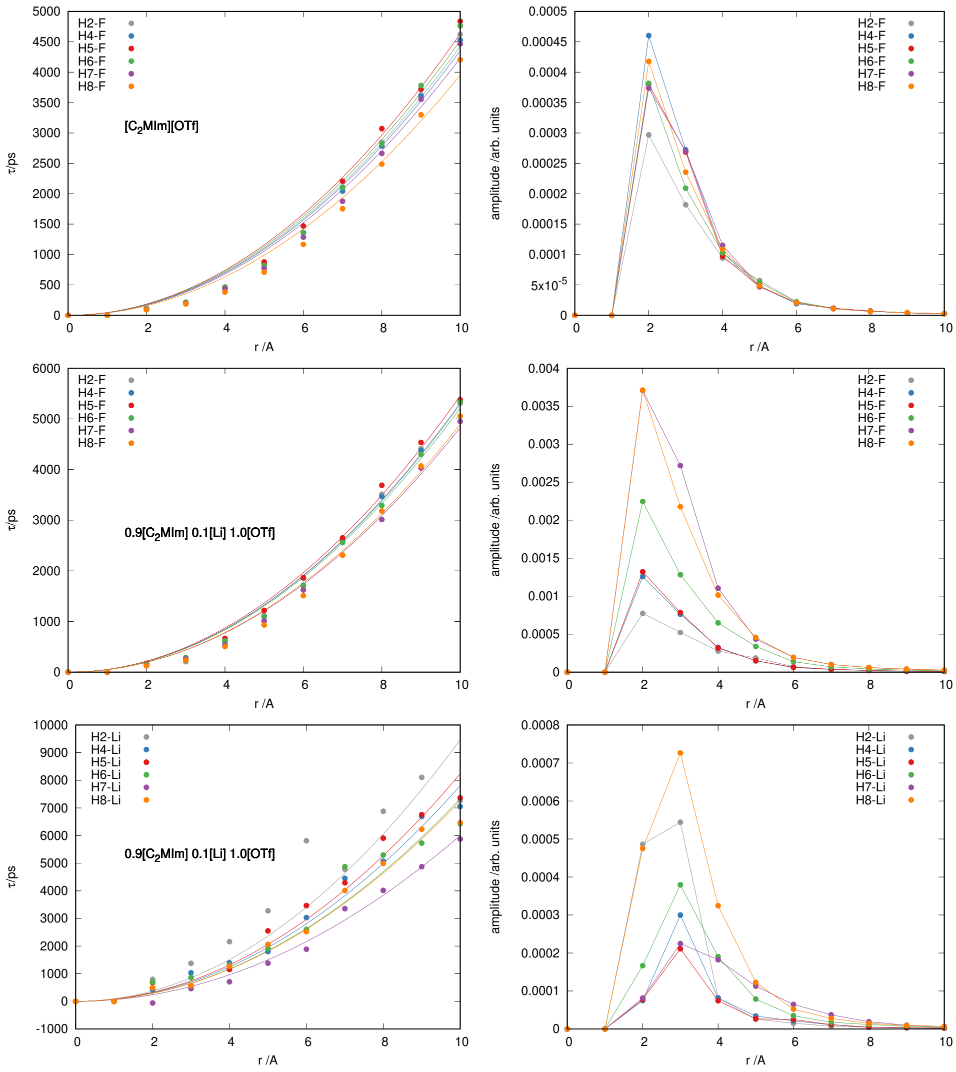

Figure 2: TCF relaxation times τ (left-hand side) and amplitudes $G(0)$ (right-hand side) of the H-F spin pairs in [C_2MIm][OTf] (top), the H-F spin pairs in 0.9[C_2MIm] 0.1[Li] 1.0[OTf] (middle) and the H-Li spin pairs in 0.9[C_2MIm] 0.1[Li] 1.0[OTf] (bottom), resolved by internuclear distance r. The solid lines in the left-hand side diagrams compare the predicted parabolic distance dependence of the relaxation time τ (ref. 37) to the relaxation times obtained directly from the trajectories.

3.3 Experimental spectra

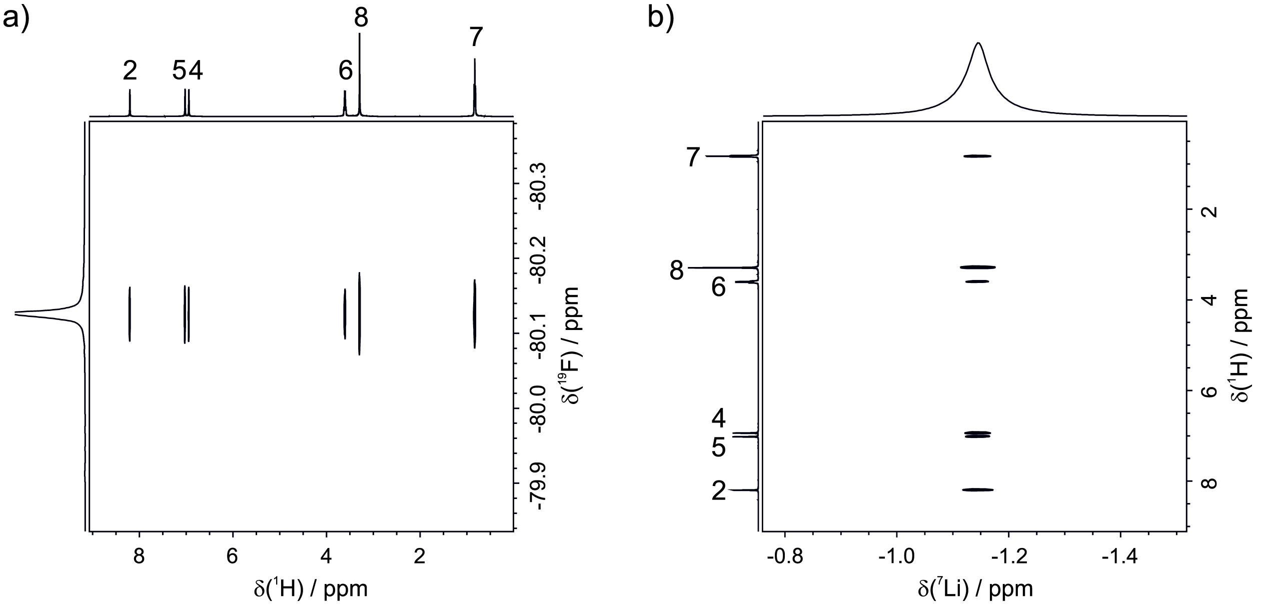

Figure 3: (a) ^{1}H-^{19}F and (b) ^{1}H-^{7}Li HOESY spectra recorded at 298 K with a mixing time of 900 ms and 1200 ms, respectively, for the mixture 0.9[C_2MIm]·0.1[Li^+]·1.0[OTf^-].

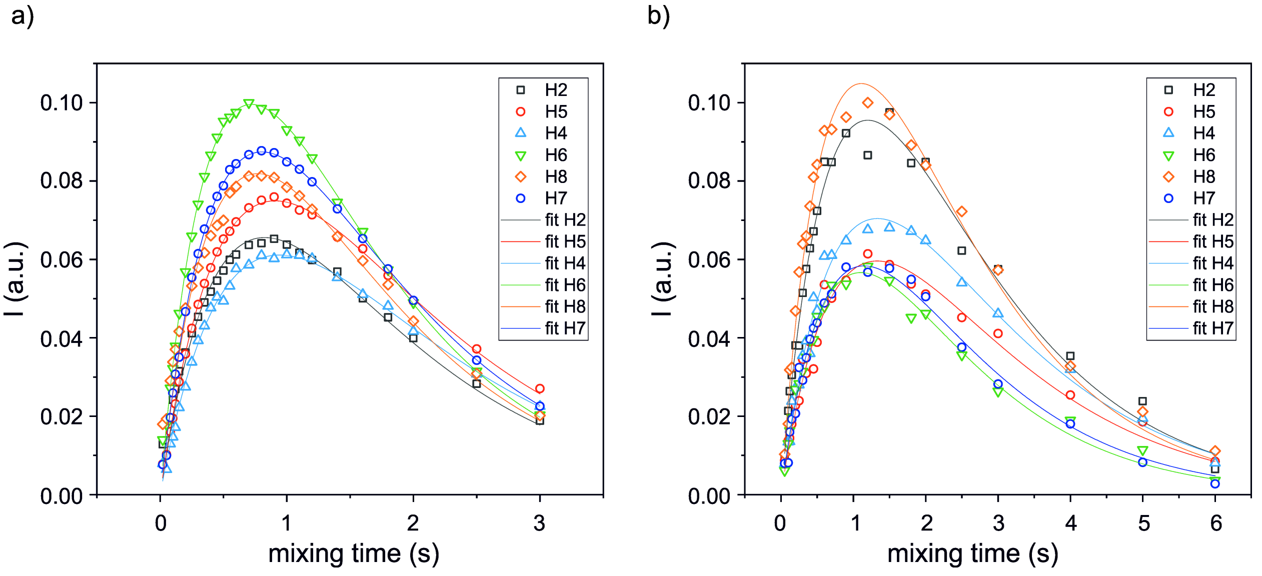

Figure 4: (a) ^{1}H-^{19}F HOESY build-up curves for the neat IL [C_2MIm][OTf^-] and corresponding fit using Eq. (2). (b) ^{1}H-^{7}Li HOESY build-up curves for the mixture 0.9 [C_2MIm] · 0.1 [Li^+] · 1.0 [OTf^-] and corresponding fit using Eq. (2).

3.4 Spatially resolved computational NOEs

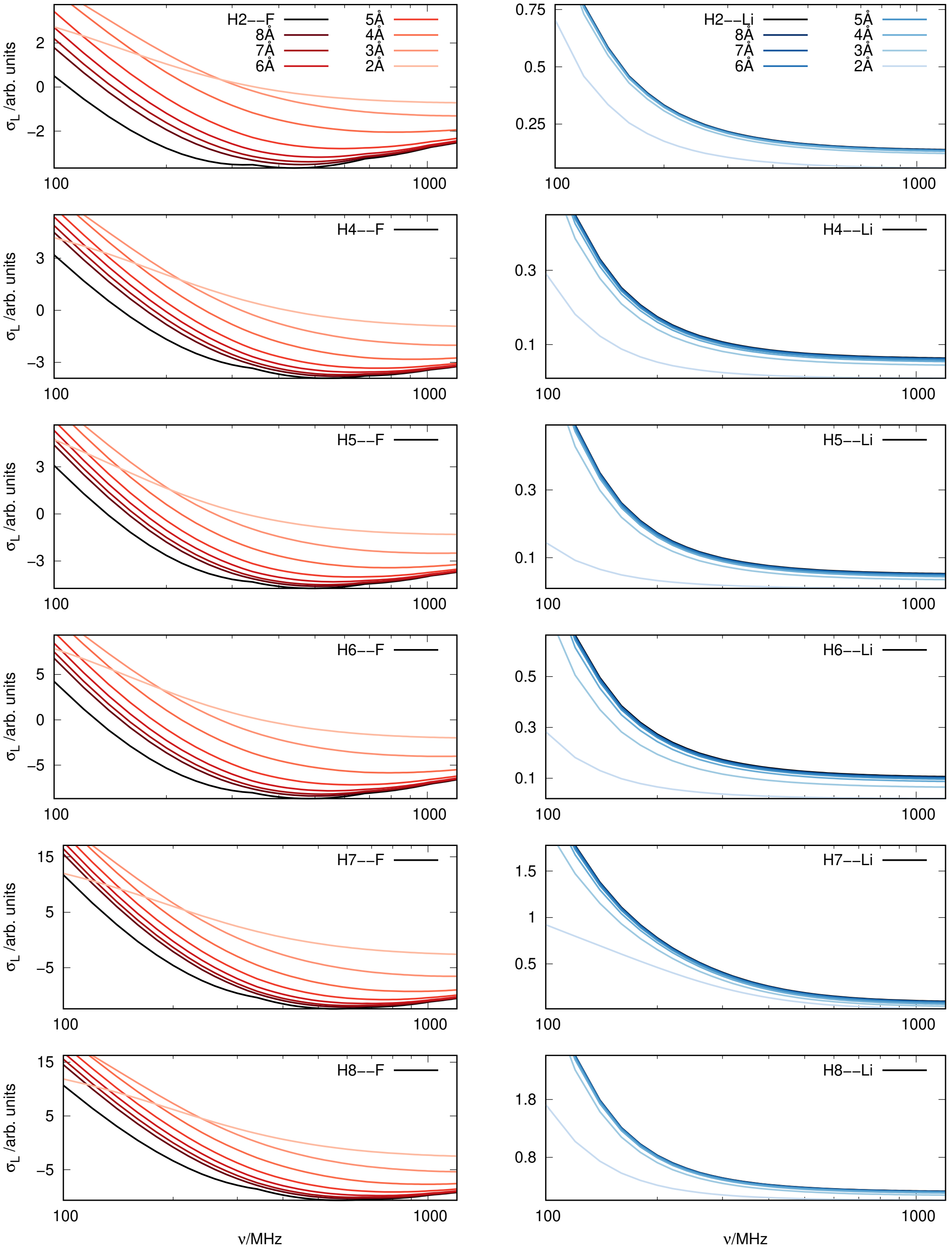

Figure 5: Cumulative contributions of shell-resolved cross-relaxation rates $\sigma_L^{\mathrm{NOESY}}(r,\nu)$ of the C_2MIm^+-F spin pairs (left-hand side) and the C_2MIm^+-Li spin pairs (right-hand side). Note that σ_L converges faster for ^{1}H-^{7}Li spin pairs than ^{1}H-^{19}F spin pairs.

References

(1) Solomon, I. Relaxation Processes in a System of Two Spins. *Phys. Rev.* **1955**, *99*, 559.

(2) Redfield, A. G. On the Theory of Relaxation Processes. *IBM J. Res. Dev.* **1957**, *1*, 19–31.

(3) Braun, D.; Schmollngruber, M.; Steinhauser, O. Revival of the Intermolecular Nuclear Overhauser Effect for Mapping Local Protein Hydration Dynamics. *J. Phys. Chem. Lett.* **2017**, *8*, 3421.

(4) Braun, D.; Steinhauser, O. The intermolecular NOE is strongly influenced by dynamics. *Phys. Chem. Chem. Phys.* **2015**, *17*, 8509–8517.

(5) D'Agostino, C.; Mantle, M. D.; Mullan, C. L.; Hardacre, C.; Gladden, L. F. Diffusion, Ion Pairing and Aggregation in 1-Ethyl-3-Methylimidazolium-Based Ionic Liquids Studied by 1H and 19F PFG NMR: Effect of Temperature, Anion and Glucose Dissolution. *ChemPhysChem* **2018**, *19*, 1081–1088.

(6) Castiglione, F.; Appetecchi, G. B.; Passerini, S.; Panzeri, W.; Indelicato, S.; Mele, A. Multiple points of view of heteronuclear NOE: Long range vs short range contacts in pyrrolidinium based ionic liquids in the presence of Li salts. *J. Mol. Liquids* **2015**, *210*, 215–222.

(7) Hou, S.-S.; Tzeng, J.-K.; Chuang, M.-H. Intermolecular association and supramolecular structures of PNVF–LiPFN and PVP–LiPFN complexes in the aqueous phase. *Soft Matter* **2010**, *6*, 409–415.

(8) Khatun, S.; Castner Jr, E. W. Ionic liquid–solute interactions studied by 2D NOE NMR spectroscopy. *J. Phys. Chem. B* **2015**, *119*, 9225–9235.

(9) Castiglione, F.; Moreno, M.; Raos, G.; Famulari, A.; Mele, A.; Appetecchi, G. B.; Passerini, S. Structural organization and transport properties of novel pyrrolidinium-

based ionic liquids with perfluoroalkyl sulfonylimide anions. *J. Phys. Chem. B* **2009**, *113*, 10750–10759.

(10) Lee, H. Y.; Shirota, H.; Castner Jr, E. W. Differences in ion interactions for isoelectronic ionic liquid homologs. *J. Phys. Chem. Lett.* **2013**, *4*, 1477–1483.

(11) Zuccaccia, C.; Bellachioma, G.; Cardaci, G.; Macchioni, A. Solution structure investigation of Ru (II) complex ion pairs: Quantitative NOE measurements and determination of average interionic distances. *J. Am. Chem. Soc.* **2001**, *123*, 11020–11028.

(12) Di Pietro, M. E.; Castiglione, F.; Mele, A. Anions as Dynamic Probes for Ionic Liquid Mixtures. *J. Phys. Chem. B* **2020**, *124*, 2879–2891.

(13) Bedrov, D.; Piquemal, J.-P.; Borodin, O.; MacKerell, A.; Roux, B.; Schröder, C. Molecular Dynamics Simulations of Ionic Liquids and Electrolytes Using Polarizable Force Fields. *Chem. Rev.* **2019**, *119*, 7940–7995.

(14) Taylor, T.; Schmollngruber, M.; Schröder, C.; Steinhauser, O. The effect of Thole functions on the simulation of ionic liquids with point induced dipoles at various densities. *J. Chem. Phys.* **2013**, *138*, 204119.

(15) Schmollngruber, M.; Lesch, V.; Schröder, C.; Heuer, A.; Steinhauser, O. Comparing induced point-dipoles and Drude oscillators. *Phys. Chem. Chem. Phys.* **2014**, *17*, 14297–14306.

(16) Heid, E.; Docampo-Alvarez, B.; Varela, L. M.; Prosenz, K.; Steinhauser, O.; Schröder, C. Langevin behavior of the dielectric decrement in ionic liquid water mixtures. *Phys. Chem. Chem. Phys.* **2018**, *20*, 15106.

(17) Huang, L.; Roux, B. Automated Force Field Parameterization for Nonpolarizable and Polarizable Atomic Models Based on Ab Initio Target Data. *J. Chem. Theory Comput* **2013**, *9*, 3543–3556.

(18) Vanommeslaeghe, K.; MacKerell Jr., A. D. Automation of the CHARMM General Force Field (CGenFF) I: Bond Perception and Atom Typing. *J. Chem. Inf. Model.* **2012**, *52*, 3144–3154.

(19) Vanommeslaeghe, K.; MacKerell Jr., A. D. Automation of the CHARMM General Force Field (CGenFF) II: Assignment of Bonded Parameters and Partial Atomic Charges. *J. Chem. Inf. Model.* **2012**, *52*, 3155–3168.

(20) Becker, T. M.; Dubbeldam, D.; Lin, L.-C.; Vlugt, T. J. H. Investigating polarization effects of CO_2 adsorption in MgMOF-74. *J. Comput. Sci.* **2016**, *15*, 86–94.

(21) Honegger, P.; Overbeck, V.; Strate, A.; Appelhagen, A.; Sappl, M.; Heid, E.; Schröder, C.; Ludwig, R.; Steinhauser, O. Understanding the Nature of Nuclear Magnetic Resonance Relaxation by Means of Fast-Field-Cycling Relaxometry and Molecular Dynamics Simulations—The Validity of Relaxation Models. *J. Phys. Chem. Lett.* **2020**, *11*, 2165.

(22) Goloviznina, K.; Canongia Lopes, J. N.; Costa Gomes, M.; Pádua, A. A Transferable, Polarisable Force Field for Ionic Liquids. *chemrxiv* **2019**, DOI: 10.26434/chemrxiv.8845526.v2.

(23) Brooks, B. R.; Brooks III., C. L.; MacKerell Jr., A. D.; Nilsson, L.; Petrella, R. J.; Roux, B.; Won, Y.; Archontis, G.; Bartels, C.; Boresch, S. et al. CHARMM: The biomolecular simulation program. *J. Comput. Chem.* **2009**, *30*, 1545.

(24) Martinez, L.; Andrade, R.; Birgin, E. G.; Martinez, J. M. Packmol: A package for building initial configurations for molecular dynamics simulations. *J. Comput. Chem.* **2009**, *30(13)*, 2157–2164.

(25) Nose, S. A unified formulation of the constant temperature molecular dynamics methods. *J. Chem. Phys.* **1984**, *81*, 511.

(26) Hoover, W. G. Canonical dynamics: Equilibrium phase-space distributions. *Phys. Rev.* **1985**, *A31*, 1695.

(27) Darden, T.; York, D.; Pedersen, L. Particle mesh Ewald: An N·log(N) method for Ewald sums in large systems. *J. Chem. Phys.* **1993**, *98*, 10089.

(28) Essmann, U.; Perera, L.; Berkowitz, M. L.; Darden, T.; Lee, H.; Pedersen, L. A smooth particle mesh Ewald method. *J. Chem. Phys.* **1995**, *103*, 8577.

(29) Michaud-Agrawal, N.; Denning, E. J.; Woolf, T. B.; Beckstein, O. MDAnalysis: A Toolkit for the Analysis of Molecular Dynamics Simulations. *J. Comput. Chem.* **2011**, *32*, 2319–2327.

(30) Gowers, R. J.; Linke, M.; Barnoud, J.; Reddy, T. J. E.; Melo, M. N.; Seyler, S. L.; Dotson, D. L.; Domanski, J.; Buchoux, S.; Kenney, I. M. et al. MDAnalysis: A Python package for the rapid analysis of molecular dynamics simulations. *Proceedings of the 15th Python in Science Conference* **2016**, 102–109.

(31) Rycroft, C. H. Voro++: A three-dimensional Voronoi cell library in C++. *Chaos* **2009**, *19*, 041111.

(32) Voronoi, G. *Journal fuer die reine and angewandte Mathematik* **1908**, *134*, 198.

(33) Brostow, W.; Castano, V. M. Voronoi polyhedra as a tool for dealing with spatial structures of amorphous solids, liquids and dense gases. *J. Mater. Educ.* **1999**, *21*, 297–304.

(34) Okabe, A.; Boots, B.; Sugihara, K.; Chiu, S. N. *Spatial tessellations: concepts and applications of Voronoi diagrams*; 1992.

(35) van Meel, J. A.; Filion, L.; Valeriani, C.; Frenkel, D. A parameter-free, solid-angle based, nearest-neighbor algorithm. *J. Chem. Phys.* **2012**, *136*, 234107.

(36) Zeindlhofer, V.; Berger, M.; Steinhauser, O.; Schröder, C. A shell-resolved analysis of preferential solvation of coffee ingredients in aqueous mixtures of the ionic liquid 1-ethyl-3-methylimidazolium acetate. *J. Chem. Phys.* **2018**, *148*, 193819.

(37) Halle, B. Cross-relaxation between macromolecular and solvent spins: The role of long-range dipole couplings. *J. Chem. Phys.* **2003**, *119*, 12373.
